# Playground Safety Assessment by Playground Rating System (PRS) Method in Qom, Iran

**Published:** 2019-08

**Authors:** Alireza Koohpaei, Mohsen Sadeghi Yarandi, Faezeh Javadi, Ali Ebrahimi, Alireza Mashkoori, Mohammad Khandan

**Affiliations:** ^*a*^Occupational Health Department, Health Faculty, Qom University of Medical Sciences, Qom, Iran.

## Abstract

**Background:**

Playing is one of the first children s activities and one of the factors affecting the health and physical and mental development of children. So have a safe park is one of the most basic rights of children. Without any doubt playgrounds should be healthy and safe environment for the children, that children can play freely and enjoy the game and to gain new experiences were. This study aiming at Playground Safety Assessment in Qom City by Playground Rating System (PRS) Method in 2017-2018 was conducted.

**Methods:**

In this cross-sectional study, 98 Park has been evaluated in Qom city. In order to evaluation the safety of playground, after assessing the validity and reliability of Playground Rating System method, mentioned method was used. Data were analyzed by using Independent sample t-test, correlation coefficient, Analysis of variance (ANOVA), Chi-Square statistical tests with SPSS software version 20. Finally, with regard to safety levels of playground, GIS maps for the playgrounds safety in the Qom City were drawn.

**Results:**

The results of this study showed that the mean and standard deviation scores of the parks studied in PRS method is a low and equal to 39.17 and 6.13 respectively. Results related to the frequency of each of the safety levels of PRS method indicated that all playground examined in this study had a total score of less than 59 and stay on “F” risk level or unsafe. The Important factor in lowering the level of safety in the playground of Qom city is how to play the game / social ergonomics. Also there was a meaningful relationship between the part of the Maintenance and safety in the PRS method with the level of incomes in various urban areas.

**Conclusions:**

The result has shown that all of playgrounds in Qom city are unsafe or has a low safety level and the level of incomes is an important factor in the safety of playground. So considering that playgrounds play an important role in the growth and development of children s physical and mental status and also the dynamics of their cities, especially in cities such as Qom, that due to the holy shrine, is a crowded city, taking corrective actions in order to making safe playground is a top priority.

**Keywords:**

Safety, Playground, Playground Rating System (PRS), Qom City

